# Diagnostic Challenge: Angiomatoid Fibrous Histiocytoma with EWSR1 Rearrangement

**DOI:** 10.3390/diagnostics16172785

**Published:** 2026-08-30

**Authors:** Tomonori Kawasaki, Jiro Ichikawa, Satoshi Kanno, Tomoaki Torigoe, Takuya Watanabe, Masataka Hirasaki, Masanori Wako, Tetsuhiro Hagino, Rikito Tatsuno, Kojiro Onohara, Atsushi Enomoto, Takayuki Nojima

**Affiliations:** 1 Department of Pathology, Saitama Medical University International Medical Center, Hidaka 350-1298, Saitama, Japan; tomo.kawasaki.14@gmail.com (T.K.); kanno820@saitama-med.ac.jp (S.K.); 2 Department of Orthopaedic Surgery, Interdisciplinary Graduate School of Medicine, University of Yamanashi, Chuo 409-3898, Yamanashi, Japan; wako@yamanashi.ac.jp (M.W.); t-hagino@yamanashi.ac.jp (T.H.); rtatsuno@yamanashi.ac.jp (R.T.); 3 Department of Orthopaedic Oncology and Surgery, Saitama Medical University International Medical Center, Hidaka 350-1298, Saitama, Japan; ttorigoe@saitama-med.ac.jp (T.T.); tw52831@5931.saitama-med.ac.jp (T.W.); 4 Department of Clinical Cancer Genomics, Saitama Medical University International Medical Center, Hidaka 350-1298, Saitama, Japan; hirasaki@saitama-med.ac.jp; 5 Department of Diagnostic Radiology, Interdisciplinary Graduate School of Medicine, University of Yamanashi, Chuo 409-3898, Yamanashi, Japan; konohara@yamanashi.ac.jp; 6 Department of Pathology, Nagoya University Graduate School of Medicine, Nagoya 466-8550, Aichi, Japan; enomoto.atsushi.d3@f.mail.nagoya-u.ac.jp; 7 Department of Pathology and Laboratory Medicine, Kanazawa Medical University, Kahoku 920-0293, Ishikawa, Japan; nojima@kanazawa-med.ac.jp

**Keywords:** angiomatoid fibrous histiocytoma, imaging, histopathology, differential diagnosis

## Abstract

This image report aims to illustrate the diagnostic value of integrating various diagnostic modalities to distinguish angiomatoid fibrous histiocytoma (AFH) from malignant soft tissue tumors. A 33-year-old man presented with a painful palpable mass in the medial left thigh. Magnetic resonance imaging revealed a 27 × 23 × 20 mm intramuscular lesion in the vastus medialis with a mixed T1 signal, predominantly high T2/STIR signal, internal hyperintense foci, a capsule-like rim, septations, heterogeneous enhancement, and marked peritumoral edema extending beyond the apparent tumor margins. Because these findings raised concern for a malignant soft tissue tumor, wide excision was performed after biopsy suggestive of low-grade sarcoma. Histologically, the tumor comprised spindle to epithelioid cells arranged in fascicular and storiform patterns surrounded by a lymphoid cuff. Immunohistochemistry showed positivity for CD68, CD99, and EMA, partial desmin and S-100 expression, and a Ki-67 index of 15%. Differential diagnosis included AFH and a malignant peripheral nerve sheath tumor. FISH demonstrated EWSR1 rearrangement, while PCR testing for EWSR1–CREB1 and EWSR1–ATF1 was negative. Although the specific fusion partner could not be identified, an EWSR1 rearrangement, together with the histologic and immunohistochemical findings, was considered supportive but not definitive for AFH. AFH, a rare intermediate tumor with nonspecific imaging features and variable pathology, makes diagnosis challenging. This case emphasizes the need to consider AFH in the diagnosis of intramuscular tumors with disproportionate peritumoral edema and highlights the role of molecular analysis in confirming the diagnosis.

**Figure 1:**
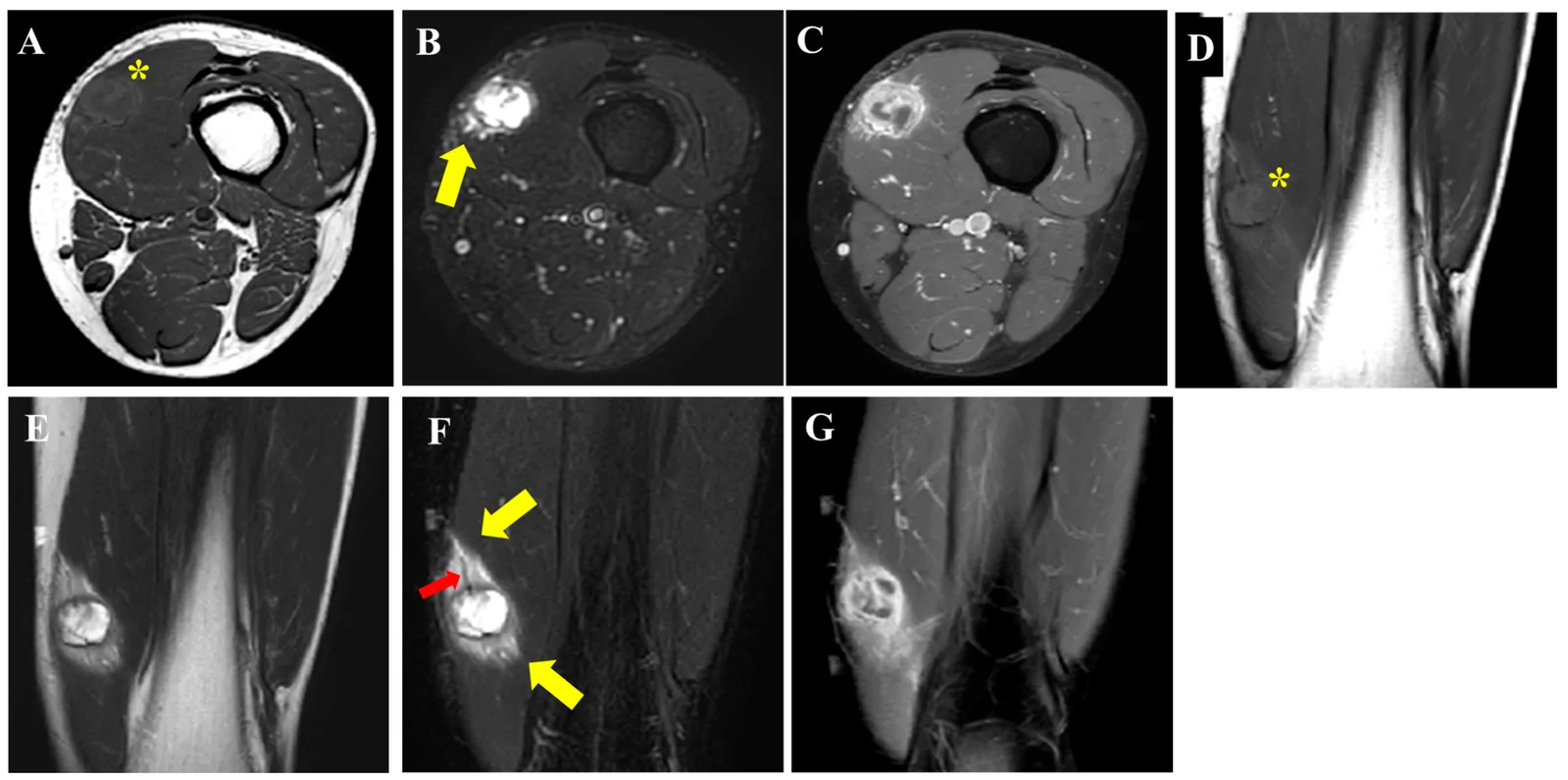
A 33-year-old man presented with pain and a palpable mass on the medial aspect of his left thigh, first noted 1 month before his initial visit to our hospital. He initially consulted a local clinic and was subsequently referred to our institution for further evaluation. Physical examination revealed spontaneous pain without cutaneous erythema or swelling. Tinel’s sign was negative, and the range of motion was preserved. His medical history was notable for irritable bowel syndrome, for which he was receiving medical treatment. Magnetic resonance imaging (MRI) demonstrated a lesion within the left vastus medialis muscle, measuring 27 × 23 × 20 mm. On T1-weighted imaging (**A**,**D**), the mass (asterisk) shows mixed iso- to slightly high signal intensity relative to that of the muscle. On T2-weighted (**E**) and STIR images (**B**,**F**), it demonstrates a predominantly high signal intensity with a markedly hyperintense area internally, along with a capsule-like rim and septum-like low-signal structures. On fat-suppressed contrast-enhanced T1-weighted images (**C**,**G**), the markedly hyperintense area on STIR shows little enhancement, whereas the remaining areas exhibit heterogeneous enhancement. In addition, signal changes extending along the surrounding muscle fiber orientation—appearing as a faintly high signal on T1-weighted images, high signal on STIR, and enhancement (yellow arrow)—are observed and extend beyond the apparent size of the mass. Dilated vascular structures are also observable adjacent to and traversing the area near the lesion (red arrow). Overall, the imaging findings suggest an intramuscular mass with cystic or hemorrhagic components and a capsule-like structure, with a pronounced surrounding tissue reaction. Based on these findings, a malignant soft tissue tumor was considered the primary differential diagnosis. A needle biopsy was therefore performed, which suggested a low-grade sarcoma. Considering the MRI and histopathological findings, a wide surgical excision was subsequently performed.

**Figure 2:**
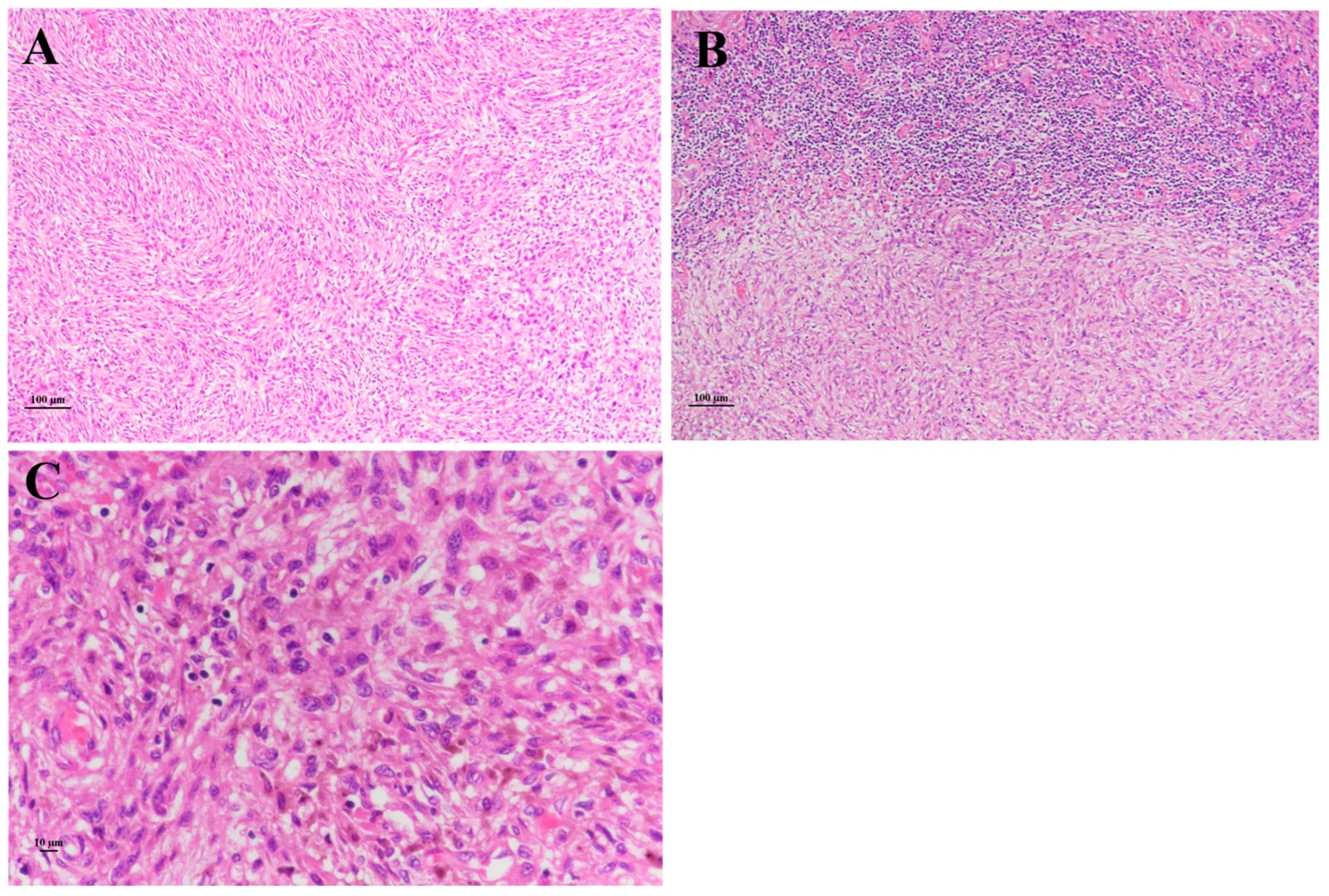
Histologically, the tumor is composed of spindle-shaped cells arranged in fascicular and storiform patterns ((**A**), ×100). A rim of lymphoid tissue with follicle formation, consistent with a lymphoid cuff, surrounds the tumor ((**B**), ×100). The tumor cells range from epithelioid to spindle-shaped and show histiocyte-like features with hemosiderin deposition in some areas. The nuclei have variable sizes and irregular contours but show no marked increase in chromatin ((**C**), ×400). Mitotic figures are scattered.

**Figure 3:**
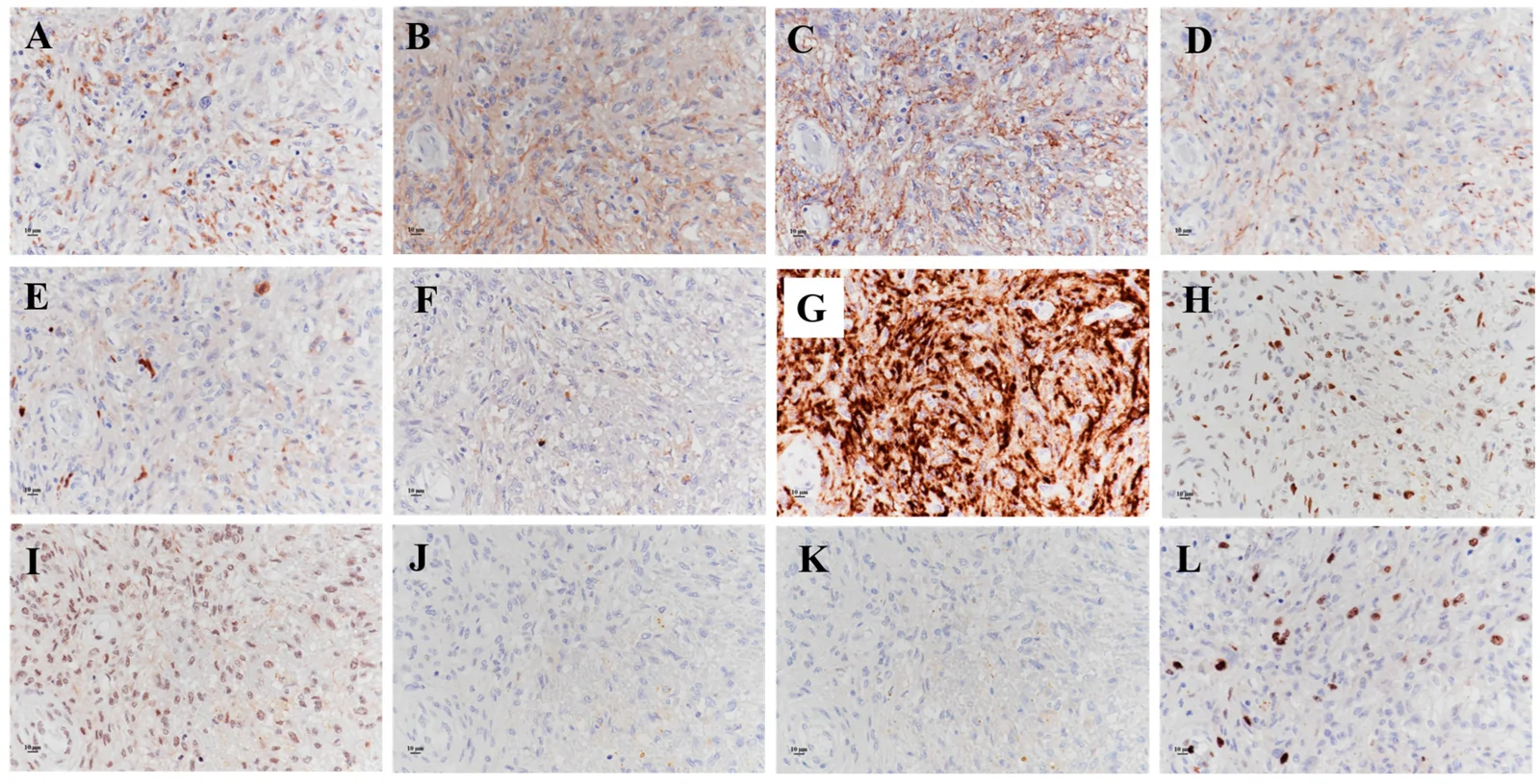
Immunohistochemically, the tumor cells are positive for CD68 ((**A**), ×400), CD99 ((**B**), ×400), EMA ((**C**), ×400), and ALK ((**D**), ×400); are partially positive for desmin ((**E**), ×400), S-100 ((**F**), ×400), and AE1AE3 ((**G**), ×400); retain positivity for H3K27me ((**H**), ×400) and INI1 ((**I**), ×400); and are negative for myogenin ((**J**), ×400) and MyoD1 ((**K**), ×400). The Ki-67 (MIB-1) labeling index is approximately 15% ((**L**), ×400). Based on these findings, angiomatoid fibrous histiocytoma (AFH) and malignant peripheral nerve sheath tumor (MPNST) were considered in the differential diagnosis. The surgical margins were negative.

**Figure 4:**
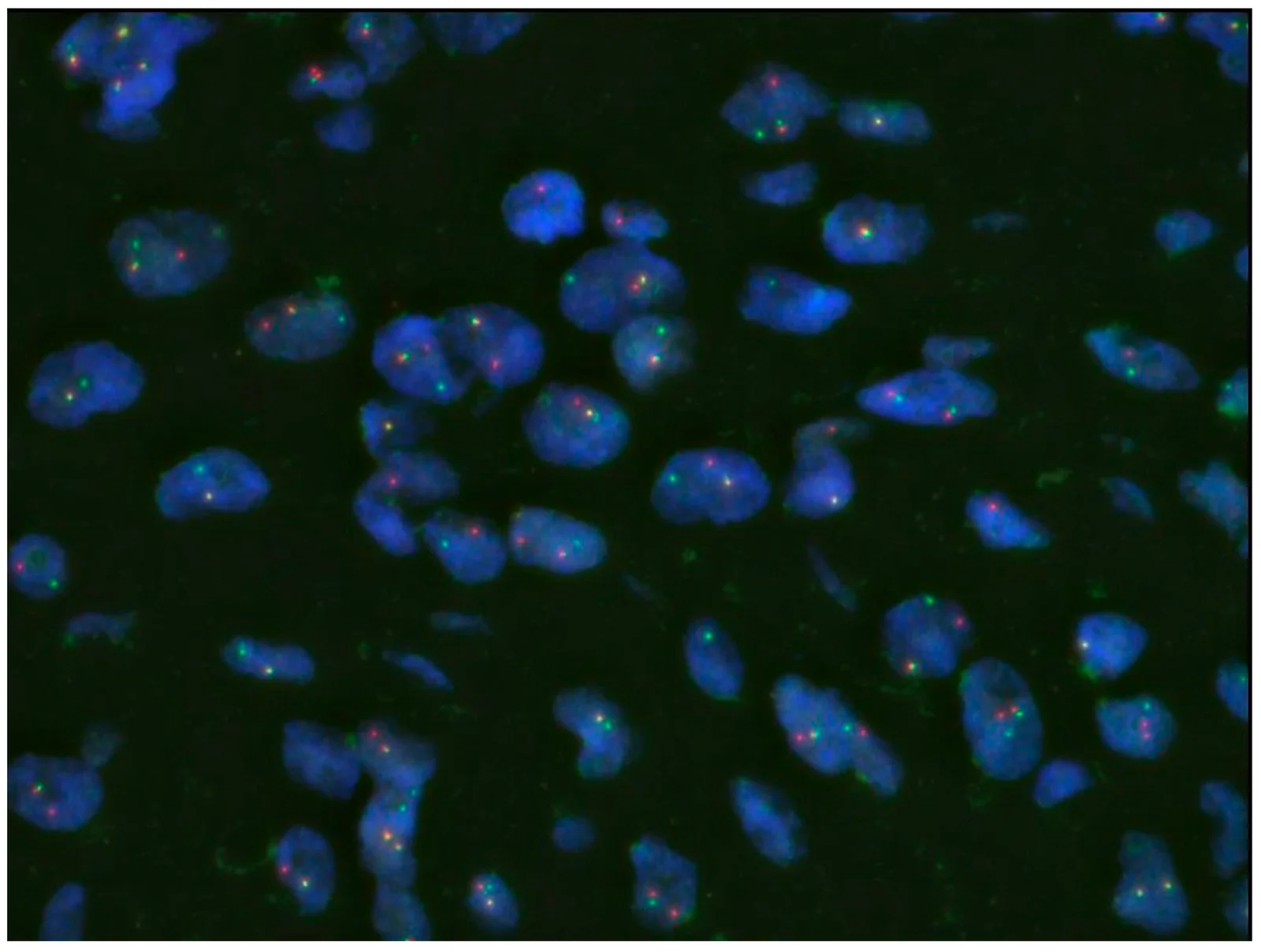
A split EWSR1 signal is detected. Further analysis of fusion partners was performed, including PCR for EWSR1–CREB1 and EWSR1–ATF1, but neither was identified. Taken together, although an EWSR1 rearrangement was present, the findings were consistent with angiomatoid fibrous histiocytoma (AFH) harboring an uncommon fusion partner. There has been no recurrence or metastasis during the three-year postoperative follow-up. AFH is a rare intermediate tumor with very low metastatic potential, accounting for approximately 0.3% of all soft tissue tumors [1]. It occurs across a wide age range, with the peak incidence occurring during the first three decades of life [2]. No clear sex predilection has been reported. The most common sites are the extremities, followed by the trunk and head and neck, and approximately two-thirds of cases arise in locations associated with lymph nodes [2]. Although AFH most frequently occurs in the subcutaneous tissue, deep-seated lesions have been reported in approximately 32–43% of cases [3]. Clinically, AFH typically presents as a slowly growing mass and is often painless, sometimes accompanied by hematoma- or hemangioma-like skin changes [1]. Reported tumor sizes range widely from 0.7 to 12 cm, with a mean size of approximately 2 cm [1]. In the present case, pain and the deep intramuscular location represented somewhat atypical features. Characteristic MRI findings of AFH include a multilocular appearance, a pseudocapsule, peritumoral edema, fluid–fluid levels, and contrast enhancement [4]. However, these findings are not specific and are also seen in other benign and malignant soft tissue tumors, such as synovial sarcomas [5], making it difficult to establish a definitive diagnosis of AFH based on MRI alone. Furthermore, because AFH lesions are often small, these characteristic features may be subtle or absent in some cases [6]. Previous reports have shown that the MRI-based differential diagnosis is broad and includes both benign and malignant soft tissue tumors; therefore, imaging alone is generally insufficient for diagnosis. Furthermore, although a small size and an indolent clinical course may suggest benignity, sarcomas can occasionally present with similar features [7], warranting careful diagnostic evaluation. Taken together, AFH should be considered in the differential diagnosis of an intramuscular mass showing disproportionately prominent peritumoral edema or inflammatory changes relative to its size. AFH exhibits a wide morphological spectrum ranging from myxoid to fibrous patterns, and the absence of a specific immunohistochemical marker often makes the histopathological diagnosis challenging [2]. The differential diagnosis is broad, encompassing reactive lesions, such as hematoma and granuloma, as well as both benign and malignant tumors. Among malignant entities, Ewing sarcoma, rhabdomyosarcoma, and malignant rhabdoid tumor should be considered [2], and misdiagnoses as myxoid liposarcoma, myxofibrosarcoma, or synovial sarcoma have been reported [4]. Ewing sarcoma requires particular diagnostic caution, as it shares a similar age distribution and commonly shows CD99 positivity and EWSR1 rearrangement [2]. Molecular analysis using FISH and PCR for fusion-gene detection is therefore valuable for establishing the diagnosis of AFH. Three types of fusion genes, including EWSR1–CREB1, EWSR1-ATF1, and FUS-ATF1, have been reported, with EWSR1–CREB1 being predominant [8]. EWSR1 rearrangement is observed in approximately 76.5% of cases [2]. In the present case, although EWSR1 rearrangement was detected, the specific fusion partner could not be identified. However, a malignant peripheral nerve sheath tumor (MPNST), which remained in the differential diagnosis until the final stage, was considered unlikely on the basis of the overall molecular findings. From the perspective of EWSR1-rearranged tumors, Ewing sarcoma and clear cell sarcoma must be included in the differential diagnosis; however, without integrating morphological and immunohistochemical findings, there is a risk of misdiagnosis [9]. In AFH, local recurrence has been reported in approximately 15% of cases [1], suggesting that a wide excision may be an appropriate treatment strategy; however, in our case, a wide resection was performed because a low-grade sarcoma could not be excluded. There is currently no definitive evidence supporting chemotherapy or radiotherapy. The reported rate of metastasis varies, ranging from approximately 5% to 29% [1,4]. The wide variation in metastatic rates may reflect differences in diagnostic criteria for AFH, and further investigation is warranted. Metastatic sites, including the lymph nodes, lungs, liver, and brain, have been reported [2]. However, mortality related to metastasis also shows variability and ranges from 0% to 14% [4]. Taken together, surgical resection should be actively considered for resectable lesions. At present, no histopathological factors have been definitively correlated with clinical outcomes [1]. This report was limited because RNA-based NGS or anchored multiplex PCR was not performed, and the fusion partner could not be identified. This case illustrates the diagnostic challenge of intramuscular AFH, which can radiologically mimic a malignant soft tissue tumor. The lesion demonstrated atypical MRI features, while histology and FISH confirmed an EWSR1 rearrangement without an identifiable fusion partner. Complete excision with negative margins was achieved, and the patient remained disease-free at follow-up. This case underscores the importance of integrating radiologic, pathologic, and molecular assessments when evaluating deep soft tissue tumors with misleading imaging findings.

## Data Availability

The original contributions presented in this study are included in the article. Further inquiries can be directed to the corresponding author.

## Abbreviations

The following abbreviations are used in this manuscript:

AFH	Angiomatoid fibrous histiocytoma
MRI	Magnetic resonance imaging
T1WI	T1-weighted imaging
T2WI	T2-weighted imaging
STIR	Short tau inversion recovery
FISH	Fluorescence in situ hybridization
PCR	Polymerase chain reaction
EWSR1	Ewing sarcoma breakpoint region 1
CREB1	cAMP responsive element-binding protein 1
ATF1	Activating transcription factor 1
FUS	Fused in sarcoma
CD68	Cluster of differentiation 68
CD99	Cluster of differentiation 99
EMA	Epithelial membrane antigen
S-100	S-100 protein
Ki-67	Kiel 67 proliferation marker
MIB-1	Mindbomb E3 ubiquitin protein ligase 1 antibody
MPNST	Malignant peripheral nerve sheath tumor
